# Identification of symptom clusters and associated factors based on disease characteristics in patients with esophageal cancer undergoing radiotherapy or chemoradiotherapy

**DOI:** 10.3389/fmed.2026.1910508

**Published:** 2026-09-09

**Authors:** Aijuan Wang, Xiuqin Ren

**Affiliations:** Nanjing First Hospital, Nanjing Medical University, Nanjing, China

**Keywords:** associated factors, chemoradiotherapy, esophageal cancer, symptom cluster, factor analysis

## Abstract

**Objective:**

To identify symptom clusters in patients with esophageal cancer undergoing radiotherapy or chemoradiotherapy, and to analyze their associated disease-related factors.

**Methods:**

A total of 289 patients receiving radiotherapy or chemoradiotherapy for esophageal cancer were recruited from the oncology departments of a tertiary general hospital in Nanjing between December 2021 and December 2023. Data were collected using a general information questionnaire and the esophageal cancer symptom assessment scale. Exploratory factor analysis and multiple linear regression were employed for statistical analysis.

**Results:**

The most prevalent symptoms were dysphagia (73.55%), fatigue (66.52%), weight loss (66.29%), sleep disturbance (62.58%), dry mouth (61.87%), shortness of breath (58.39%), nausea (57.42%), and pruritus (57.00%). Higher symptom incidence was associated with greater severity and distress. Four distinct symptom clusters were identified: psychosomatic, dysphagia, adverse reaction, and reflux-cough. Clinical stage, lesion location, and treatment modality emerged as independent predictors of symptom cluster severity (*P* < 0.05).

**Conclusion:**

Patients with esophageal cancer undergoing radiotherapy or chemoradiotherapy experience multiple concurrent symptoms that form identifiable clusters. Clinical nursing staff should prioritize the leading symptoms within each cluster and develop targeted interventions based on symptom severity and distress levels, thereby improving symptom management effectiveness and enhancing patients’ quality of life during the treatment and recovery period.

## 1 Introduction

Global cancer statistics for 2020 reported approximately 604,000 new cases of esophageal cancer and 544,000 deaths worldwide (1). Surgery and chemoradiotherapy constitute the primary treatment modalities for this malignancy. During the postoperative chemoradiotherapy period, patients frequently experience a constellation of symptoms, including cough, dysphagia, odynophagia, nausea, vomiting, dry mouth, and reflux. These symptoms are influenced by surgical technique, anastomotic reconstruction, and the toxic effects of chemoradiation. Importantly, symptoms may interact synergistically, potentially creating a vicious cycle of escalating symptom burden. This phenomenon exerts a substantial impact on quality of life, particularly in both short-term and long-term outcomes (2–4). Clusters of symptoms tend to co-occur and evolve dynamically throughout the treatment trajectory, exerting more powerful negative effects on prognosis than individual symptoms alone (3).

Our research team has previously reported on the relationships among symptom clusters, neutrophil-to-lymphocyte ratio, albumin levels, anxiety, depression, social support, self-care, and self-efficacy in this patient population. We have also conducted qualitative research on symptom experiences and self-management strategies during chemoradiotherapy (5, 6). Although previous studies have described symptom clusters in esophageal cancer patients receiving radiotherapy and/or chemoradiotherapy, the vast majority have confined their analyses to cluster composition alone, leaving a critical gap: how disease-related characteristics—specifically lesion site, clinical stage, and treatment modality—are independently and quantitatively associated with the severity of each cluster remains largely unexamined. Furthermore, nearly all existing cluster analyses are cross-sectional and static, whereas emerging longitudinal network evidence [e.g., Sun et al. (7),] indicates that symptom interconnections are time-varying and may shift dynamically across treatment phases.

Positioning our work within this evolving landscape, the present study does not claim primacy in identifying symptom clusters. Instead, we provide a clinically annotated, factor-derived cluster model that offers two distinct contributions: (a) a practical, bedside-accessible framework for early risk stratification, and (b) a baseline empirical snapshot that can anchor and inform future longitudinal or network-based investigations into symptom dynamics during chemoradiotherapy.

## 2 Materials and methods

### 2.1 Subjects and methods

#### 2.1.1 Study participants

A convenience sampling method was employed. Patients undergoing radiotherapy or chemoradiotherapy for esophageal cancer were recruited from the oncology departments of one affiliated hospital of Nanjing Medical University between December 2021 and December 2023. The inclusion criteria were: (1) hospitalized patients receiving radical chemoradiotherapy for the first time or postoperative adjuvant/concurrent chemoradiotherapy for esophageal cancer, with a definitive pathological diagnosis. The radiation dose was 1.8–2.0 Gy per fraction, administered for ≥30 fractions. Concurrent chemotherapy regimens adhered to the *Esophageal Cancer Diagnosis and Treatment Guidelines* (2022 Edition); (2) age between 18 and 80 years; (3) provision of informed consent and voluntary participation in the survey; (4) Karnofsky Performance Status (KPS) score ≥70.

The exclusion criteria were: (1) patients with cognitive or psychiatric impairment; (2) patients with severe cardiac, pulmonary, hepatic, or renal dysfunction; (3) patients undergoing re-irradiation for recurrent esophageal cancer; (4) children aged ≤18 years, pregnant or lactating women, and patients with advanced esophageal cancer in an extremely debilitated condition; (5) instances where family members withheld disease-related information from the patient for protective reasons; (6) patients or their primary caregivers who did not have access to WeChat or were unable to use it proficiently.

The required sample size for factor analysis was calculated based on a ratio of 5 to 10 subjects per questionnaire item. The symptom assessment questionnaire comprised 31 items, leading to an estimated sample size of 155–310 cases. This study was ethically approved by the Institutional Review Board of Nanjing Hospital Affiliated to Nanjing Medical University.

### 2.2 Survey instruments

#### 2.2.1 Demographic information questionnaire

Developed by the research team through an extensive literature review and leveraging clinical experience, this questionnaire encompassed variables like gender, age, education level, medical payment method, clinical stage, lesion site, and treatment modality.

#### 2.2.2 Esophageal cancer symptom assessment scale

The scale was constructed based on the Delphi method by Hu (3) from the Fourth Hospital of Hebei Medical University, designed to assess symptoms in patients with esophageal cancer. The scale comprises 31 items, each rated on a 4-point Likert scale, with scores ranging from 1 (“rarely present”) to 4 (“persistently present”), where higher scores indicate more severe symptoms. The Cronbach's *α* coefficients of the total scale and subscales ranged from 0.722 to 0.937, with a cumulative variance contribution rate of 73.091%. The scale has been previously developed and validated for the investigation of symptom clusters in esophageal cancer patients undergoing chemoradiotherapy and demonstrates excellent reliability and validity in this patient population.

### 2.3 Data collection procedures

Trained research team members used a WeChat mini-program to collect symptom data during the middle phase of radiotherapy (fractions 10–20), referring to the 24 h period preceding the symptom survey. All investigators were certified Jiangsu Provincial Oncology Nurse Specialists with basic research skills. Standardized training was provided, and they were considered qualified before data collection commenced. Patients were informed of the study's aim and precautions before the survey began. Any questions concerning particular items were resolved. Questionnaires were administered and retrieved on-site. Patients were asked to complete missing information immediately if any items were left blank. If a patient's emotional or physical state had altered, the survey was rescheduled. Data were independently entered, verified, and collated by two researchers. A total of 310 questionnaires were distributed, yielding 289 valid responses, with an effective response rate of 93.2%.

### 2.4 Statistical analysis

Statistical analyses were performed using SPSS version 26.0. Categorical variables were presented as frequencies and percentages, while continuous variables were shown as mean ± standard deviation. Non-parametric rank sum tests were used for between-group comparisons. Multiple linear regression analysis was utilized to identify factors influencing symptom clusters. Principal component analysis, a form of exploratory factor analysis, was employed to identify the types of symptom clusters. Common factors (i.e., symptom clusters) were extracted based on the following criteria: eigenvalue ≥ 1; symptom incidence > 20%; factor loading ≥ 0.4 for symptoms within the factor. If a symptom exhibited a loading ≥ 0.4 on multiple factors, it was assigned to the factor with the highest loading. Each factor was required to contain at least two items. Symptom clusters were named according to clinical relevance and the compositional characteristics of the constituent symptoms.

## 3 Results

### 3.1 Participant characteristics

A total of 289 patients (198 males and 91 females) were included in the final analysis. The mean age was 67.77 ± 5.25 years (range: 38–84 years), with 161 patients (55.7%) older than 65 years. Additional demographic and clinical characteristics are presented in Table 1.

**Table 1:** Demographic characteristics and univariate analysis of symptom cluster (SC) during Mid-radiotherapy in esophageal cancer patients (*n* = 289).

Variable		*n* (%)	Reflux-cough SC	Dysphagia SC	Adverse reaction SC	Energy deficiency SC
Sex	Male	198 (68.5)	17.58 ± 5.85	16.35 ± 4.33	10.74 ± 3.78	15.06 ± 3.32
	Female	91 (31.5)	18.46 ± 5.49	17.33 ± 4.85	11.16 ± 3.78	15.18 ± 3.37
	Test Statistic		−1.219^2^	−1.712^2^	−0.892^2^	−0.273^2^
	*P* value		0.224	0.088	0.373	0.785
Education level	Primary School	62 (21.5)	19.18 ± 5.89	17.00 ± 4.38	11.68 ± 3.79	15.23 ± 3.39
	Middle School	148 (51.2)	17.57 ± 5.86	16.99 ± 4.63	10.72 ± 3.71	15.27 ± 3.22
	High School	48 (16.6)	17.75 ± 5.52	16.31 ± 3.95	11.38 ± 3.87	14.81 ± 3.54
	Undergraduate/Postgraduate	31 (10.7)	16.71 ± 4.97	14.97 ± 4.854	9.23 ± 3.509	14.45 ± 3.443
	Test Statistic		1.640^1^	1.938^1^	3.344^1^	0.666^1^
	*P* value		0.180	0.124	0.020	0.573
Payment method	Self-pay	45 (15.6)	18.49 ± 5.05	17.07 ± 4.545	11.33 ± 3.36	15.53 ± 3.123
	Medical Insurance	200 (69.2)	17.74 ± 5.87	16.59 ± 4.52	10.79 ± 3.85	15.02 ± 3.37
	Public Coverage	44 (15.22)	17.94 ± 5.42	16.68 ± 5.17	10.84 ± 3.87	15.24 ± 3.27
	Test Statistic		0.893^2^	0.655^2^	0.891^2^	0.956^2^
	*P* value		0.375	0.513	0.374	0.340
Clinical stage	Stage I-II	44 (15.2)	15.55 ± 5.793	15.25 ± 4.621	10.02 ± 4.100	13.43 ± 4.212
	Stage III-IV	245 (84.8)	18.27 ± 5.65	16.91 ± 4.46	11.02 ± 3.71	15.40 ± 3.06
	Test Statistic		−2.934^2^	−2.266^2^	−1.623^2^	−2.956^2^
	*P* value		0.004	0.024	0.106	0.005
Age	<55	66 (22.8)	17.35 ± 6.31	16.58 ± 4.90	10.76 ± 3.61	15.64 ± 3.280
	55–65	62 (21.5)	17.84 ± 5.74	16.44 ± 4.20	10.65 ± 3.88	15.11 ± 3.28
	>65	161 (55.7)	18.07 ± 5.52	16.78 ± 4.49	11.01 ± 3.83	14.87 ± 3.37
	Test Statistic		0.366^1^	0.146^1^	0.242^1^	1.243^1^
	*P* value		0.694	0.864	0.785	0.290
Lesion site	Cervical	28 (9.7)	21.32 ± 5.18	18.86 ± 3.08	12.96 ± 3.27	16.61 ± 2.33
	Upper Thoracic	45 (15.6)	19.29 ± 5.37	18.40 ± 4.12	12.40 ± 3.49	15.87 ± 2.92
	Middle Thoracic	129 (44.6)	17.43 ± 5.78	15.68 ± 4.77	10.50 ± 3.70	14.91 ± 3.38
	Lower Thoracic	87 (30.1)	16.62 ± 5.56	16.51 ± 4.28	9.95 ± 3.76	14.49 ± 3.56
	Test Statistic		6.224^2^	6.879^2^	7.965^2^	3.922^2^
	*P* value		0.000	0.000	0.000	0.009
Treatment mode	Radiotherapy	72 (24.9)	16.06 ± 6.35	15.17 ± 4.44	9.88 ± 3.71	14.32 ± 3.58
	Chemoradiotherapy	217 (75.1)	18.45 ± 5.42	17.16 ± 4.44	11.20 ± 3.75	15.35 ± 3.214
	Test Statistic		−2.875^2^	−3.294^2^	−2.608^2^	−2.302^2^
	*P* value		0.005	0.001	0.010	0.022

1 *F* value.

2 *t* value.

### 3.2 Prevalence and severity of common symptoms in patients undergoing esophageal cancer chemoradiotherapy

During the middle phase of radiotherapy, the incidence and severity of symptoms varied among patients. A total of 23 symptoms had an incidence exceeding 20%. The symptoms with the highest incidence rates were dysphagia (73.55%), fatigue (66.52%), weight loss (66.29%), sleep disturbance (62.58%), dry mouth (61.87%), shortness of breath (58.39%), nausea (57.42%), and pruritus (57.00%). These symptoms also exhibited higher severity scores. Detailed data are available in Reference 5 (5).

### 3.3 Exploratory factor analysis of symptom clusters(SC)

Exploratory factor analysis was performed on the 31 symptom items from the esophageal cancer-specific symptom assessment scale. Principal axis factoring (PAF) was employed as the extraction method, and Promax oblique rotation was applied to allow for correlations among the resulting factors—a reasonable assumption given that symptom clusters in cancer patients are clinically interrelated rather than mutually independent.

Prior to the final factor solution, eight items (Q12, Q17, Q22, Q2-2, Q2-3, Q2-4, Q2-5, and Q2-6) were excluded due to insufficient validity, defined as low factor loadings (<0.40) on all factors, substantial cross-loadings lacking coherent clinical interpretation, or low communality estimates (<0.30) suggesting poor representation by the extracted factor structure.

The final factor analysis was performed on the remaining 23 items. The Kaiser-Meyer-Olkin (KMO) measure was 0.946 (>0.7), and Bartlett's test of sphericity was significant (*χ*^2^ = 3,848.315, df = 253, *P* < 0.001), confirming the suitability of the data for factor analysis. The number of factors retained was determined based on eigenvalues > 1.0 (Kaiser criterion), inspection of the scree plot, and clinical interpretability. Four factors with eigenvalues > 1.0 were extracted, with initial eigenvalues of 9.894, 1.982, 1.571, and 1.323, respectively. These four factors collectively accounted for 64.217% of the total variance (43.017%, 8.617%, 6.829%, and 5.754%, respectively) (Table 2).

**Table 2:** Summary of factor analysis of symptoms among esophageal cancer patients (*n* = 289).

Symptom	Factor loading			
	Factor 1	Factor 2	Factor 3	Factor 4
Belching	0.790			
Counterflow	0.815			
Stomach burning sensation	0.805			
Cough	0.789			
Anorexia	0.747			
Feeling full after eating	0.804			
Abdominal distention	0.729			
Nausea		0.761		
Vomiting		0.717		
Constipation		0.760		
Pruritus		0.686		
Dry mouth		0.761		
Alopecia		0.722		
Difficulty swallowing			0.644	
Pain or discomfort with eating			0.686	
Pain			0.657	
Anxiety			0.740	
Sadness			0.636	
Fatigue				0.699
Shortness of breath				0.693
Disturbed sleep				0.713
Weight loss				0.672
dizziness				0.601
Eigenvalue	9.894	1.982	1.571	1.323
Variance explained (%)	43.017	8.617	6.829	5.754
Cumulative variance (%)	43.017	51.633	58.463	64.217
Cronbach's *α*	0.944	0.883	0.818	0.792

Factor 1, Reflux-cough SC; Factor 2, Adverse reaction SC; Factor 3, Dysphagia SC; Factor 4, Energy deficiency SC.

Symptoms with factor loadings ≥0.40 were retained within their respective groups; all retained items demonstrated primary loadings ranging from 0.599 to 0.815, with no clinically meaningful cross-loadings exceeding the predefined threshold. Based on the composition and clinical characteristics of the symptoms, the four clusters were labeled as follows: (a) “reflux-cough SC”, comprising belching, acid reflux, stomach burning sensation, cough, anorexia, feeling full after eating, and abdominal distention; (b) “adverse reaction SC”, comprising nausea, vomiting, constipation, pruritus, dry mouth, and alopecia; (c) “dysphagia SC”, comprising difficulty swallowing, pain or discomfort with eating, pain, anxiety, and sadness; and (d) “energy deficiency SC”, comprising fatigue, shortness of breath, disturbed sleep, weight loss, and dizziness. The internal consistency of each cluster was assessed using Cronbach's *α*, with values of 0.944, 0.883, 0.818, and 0.792 for Factors 1 through 4, respectively (Table 2).

### 3.4 Association between disease characteristics and symptom cluster (SC)

Univariate analyses were performed to examine the relationships between demographic/clinical characteristics and each symptom cluster score. As shown in Table 1, clinical stage, lesion site, and treatment modality were significantly associated with multiple symptom cluster scores (*P* < 0.05). Specifically, patients with advanced clinical stage (III–IV), cervical or upper thoracic lesions, and those receiving concurrent chemoradiotherapy tended to report higher scores across most symptom clusters. Detailed comparisons are presented in Table 1.

### 3.5 Regression analysis of symptom cluster (SC)

To explore disease-related predictors of symptom cluster severity, multiple linear regression analyses were performed. Symptom cluster scores were entered as dependent variables, and variables that reached statistical significance (*P* < 0.05) in univariate analyses were included as independent variables. Categorical variable assignments were as follows: education level: primary school or below = 1, middle school = 2, high school/vocational school = 3, college or above = 4; clinical stage: stage I–II = 1, stage III–IV = 2; age: <55 = 1, 55–65 = 2, >65 = 3; lesion site (dummy coded, with middle thoracic as reference): cervical = (1,0,0), upper thoracic = (0,1,0), lower thoracic = (0,0,1); treatment modality: radiotherapy = 1, chemoradiotherapy = 2. The dependent variables comprised scores for the reflux-cough, dysphagia, adverse reaction, and energy deficiency symptom clusters. We acknowledge that stepwise regression is associated with well-documented limitations, including model instability and overfitting risk (8). Accordingly, our results are presented as exploratory and hypothesis-generating.

The final regression models revealed distinct predictor patterns across symptom clusters. Dysphagia SC was significantly associated with cervical lesion location, treatment modality, clinical stage, and upper thoracic lesion location. Reflux-cough SC was significantly associated with upper thoracic location, and cervical location. Adverse reaction SC was significantly predicted by cervical location, upper thoracic location, and treatment modality. Energy deficiency SC was significantly associated with cervical location and clinical stage.

Notably, the adjusted *R*^2^ values across all models ranged from 0.063 to 0.104 (0.104 for reflux-cough SC, 0.092 for dysphagia SC, 0.091 for adverse reaction SC, and 0.063 for energy deficiency SC), with corresponding *F* values of 8.241, 9.650, 5.776, and 9.58, respectively. These consistently low adjusted *R*^2^ values suggest that the disease-related characteristics included in our analysis accounted for only a small fraction of the variance in symptom cluster scores. Unmeasured multidimensional factors—including inflammatory biomarkers, psychological distress, social support, and nutritional status—likely contribute substantially to symptom cluster severity and warrant investigation in future studies. The detailed regression output results are summarized in Table 3.

**Table 3:** Multiple linear regression analysis of symptom cluster (SC) in patients undergoing esophageal cancer chemoradiotherapy.

Dependent variable	Independent variable	Regression coefficient	Standard error	Standard regression coefficient	*t* value	*P* value
Reflux-cough SC	Constant	9.330	2.094		4.455	0.000
	Cervical	3.662	1.109	0.189	3.301	0.001
	Upper Thoracic	2.051	0.899	0.130	2.281	0.023
Dysphagia SC	Constant	12.825	1.069		11.998	0.000
	Treatment Mode	1.837	0.592	0.176	3.104	0.002
	Clinical Stage	2.287	0.901	0.143	2.537	0.012
	Upper Thoracic	2.391	0.709	0.192	3.373	0.001
	Cervical	2.555	0.874	0.168	2.924	0.004
Adverse reaction SC	Constant	8.242	0.895		9.210	0.000
	Cervical	2.498	0.732	0.196	3.413	0.001
	Upper Thoracic	2.121	0.594	0.204	3.573	0.000
	Treatment Mode	1.175	0.496	0.135	2.371	0.018
Energy deficiency SC	Constant	11.491	0.998		11.518	0.000
	Clinical Stage	1.873	0.531	0.202	3.524	0.000
	Cervical	1.505	0.645	0.134	2.331	0.020

Adjusted *R*^2^ values for the Reflux-cough SC, Dysphagia SC, Adverse reaction SC, and Energy deficiency SC were 0.104, 0.092, 0.091, and 0.063, respectively. Corresponding *F* values were 8.241, 9.650, 5.776, and 9.58.

## 4 Discussion

### 4.1 Multiple symptom clusters(SC) exist in patients undergoing esophageal cancer chemoradiotherapy

A total of 289 patients were sampled at the mid-phase of chemoradiotherapy. Factor analysis identified four symptom clusters with a total variance contribution rate of 64.217%.

1. The reflux-cough SC includes acid reflux, cough, heartburn, belching, anorexia, postprandial fullness, and abdominal distension. In this group, 59.13% of patients reported esophageal reflux symptoms. This is attributable to the removal of the physiological anti-reflux barrier during surgery and the exposure of the remaining stomach to negative intrathoracic pressure (9–11). Chronic reflux predisposes patients to heartburn, acid regurgitation, belching, and pulmonary inflammation, which can manifest as chronic cough. Moreover, esophagectomy typically involves gastric pull-up reconstruction, which substantially reduces gastric volume and capacity. Patients consequently experience early satiety and abdominal bloating, culminating in reduced appetite (12). From these findings, two symptom management pathways are recommended: (1) targeted pharmacological therapy combined with comprehensive lifestyle modifications, including elevation of the head of the bed, avoidance of large fatty meals before bedtime, and moderate post-meal activity; and (2) interventions that incorporate family support, social support, and individual behavioral strategies (13, 14).
2. The dysphagia SC encompasses dysphagia, odynophagia, pain, anxiety, and sadness. Dysphagia was present in 73.55% of cases. In our cohort, 84.8% of patients were at advanced stages and were typically ineligible for surgical resection, already experiencing swallowing difficulties that often restricted them to a semi-liquid diet. Patients receiving concurrent chemotherapy have been reported to have a 4.019-fold higher risk of developing radiation esophagitis than those receiving radiotherapy alone (8). During the middle-to-late phase of chemoradiotherapy, cumulative radiation doses of 20 Gy begin to damage the esophageal mucosa, causing odynophagia. At 40 Gy, decreased thickness of the smooth muscle folds and submucosal fibrosis further worsen dysphagia, which correlates strongly with the patient's interest in food and eating; some patients may ultimately become unable to tolerate oral intake (15). Chronic eating difficulties can raise doubts about treatment effectiveness and erode confidence in therapeutic outcomes, leading to anxiety and depression regarding the future. Affective or psychological symptom clusters have been identified as among the most common across different cancer types and disease stages in prior studies (16). Therefore, careful monitoring of dysphagia during chemoradiotherapy is essential, along with timely pharmacological and non-pharmacological interventions and enhanced psychological support.
3. The adverse reaction SC comprises constipation, nausea, vomiting, dry mouth, pruritus, and alopecia. In this study, 75.1% of patients received concurrent chemoradiotherapy. Chemotherapeutic agents, such as paclitaxel and platinum compounds, as well as antiemetics and antihistamines, may increase the risk of constipation (17). The incidence of dry mouth was 61.87%, mostly attributable to radiation fields encompassing the cervical or upper thoracic region. Radiation can also affect the oral cavity and salivary glands, reducing mucosal cell proliferation and causing salivary gland damage, resulting in reduced saliva and xerostomia (17). Cutaneous changes within the irradiated target volume—including epidermal atrophy, soft tissue fibrosis, and telangiectasia—may cause mild pruritus (18). Alopecia is a common mild side effect of chemoradiotherapy. Healthcare professionals are encouraged to adhere to clinical practice guidelines for the prevention and management of radiation-induced injury, utilizing both non-pharmacological and pharmacological approaches, to help patients mitigate these symptoms, complete their chemoradiotherapy regimen successfully (2), and reduce symptom-related distress and burden.
4. The energy deficiency SC includes fatigue, sleep disturbance, shortness of breath, dizziness, and weight loss. Most patients in this study were elderly, prone to fatigue, dizziness, and dyspnea due to sleep deprivation and physical deconditioning during chemoradiotherapy. This is consistent with findings reported by Yu et al. (2). Fatigue and related symptoms are thought to be mediated by pro-inflammatory cytokines (IL-1, IL-6, TNF-α, and IFN-α) induced by chemoradiotherapy, along with immune dysfunction, anemia, and mitochondrial dysfunction (19). Additional health education is needed to inform patients that this symptom cluster may develop during chemoradiotherapy and can persist beyond treatment completion. It is important to emphasize that these symptoms are normal treatment responses rather than indicators of treatment failure or disease progression. Regular, individualized exercise tailored to the patient's functional level can facilitate physical recovery and alleviate symptoms such as fatigue, insomnia, and sleep disturbances (20).

### 4.2 Analysis of factors influencing symptom clusters (SC) in patients undergoing esophageal cancer chemoradiotherapy

#### 4.2.1 Lesion site is an influencing factor for symptom cluster (SC)

As shown in Table 3, univariate analysis revealed significant differences in the scores of the reflux-cough, dysphagia, adverse reaction, and energy deficiency SCs across different lesion sites (*P* < 0.01). Multiple linear regression further identified cervical and upper thoracic location as important predictors of symptom cluster severity (Table 3). Patients with cervical and upper thoracic esophageal cancer tend to develop symptoms such as odynophagia, worsening dysphagia, acid reflux, burning sensation, nausea, vomiting, decreased appetite, and weight loss within 2–3 weeks of chemoradiotherapy initiation. Post-treatment symptoms, particularly odynophagia and dysphagia, are more pronounced than in tumors at other locations. Changes in food quality and reduced oral intake lead to gradual weight decline. The severity and distress of radiation-induced esophagitis and pneumonitis—characterized by reflux, cough, dyspnea, nausea, and vomiting—progressively intensify, making the symptom experience increasingly noticeable (21–25). In contrast, patients with middle and lower thoracic esophageal cancer are more likely to experience moderate-to-severe esophageal reflux during the mid-phase of chemoradiotherapy, attributable to loss of the anti-reflux barrier and exposure of the stomach to negative intrathoracic pressure. In such patients, symptoms of the reflux-cough SC, such as anxiety, are more likely to occur (22, 23). Therefore, clinical nursing staff should conduct dynamic symptom assessments based on lesion location, anticipate the emergence of symptom clusters, and recommend a diet of warm, non-irritating, semi-liquid foods with adequate fluid intake. For patients experiencing moderate-to-severe pain that compromises oral intake, interventions may include intravenous fluid administration, anti-inflammatory agents, corticosteroids, acid-suppressive therapy, and oral mucosal protectants such as sucralfate. Judicious use of analgesics and non-pharmacological interventions can further enhance patients' symptom rehabilitation and self-management capacity.

#### 4.2.2 Treatment modality is an influencing factor for symptom clusters

Univariate analysis demonstrated statistically significant differences across all four symptom clusters based on treatment modality (*P* < 0.01). Multiple linear regression confirmed that treatment modality is an influencing factor for both the adverse reaction and dysphagia SCs, consistent with the results of a study published in 2021 (26). Advanced disease stage was found in 84.8% of our cohort. Concurrent chemoradiotherapy can improve short-term outcomes and may show a possible trend toward better long-term survival in this patient group (27, 28), but it also increases the cytotoxic effects of chemotherapy. The risk of developing radiation esophagitis is 4.019-fold higher than with radiotherapy alone (29, 30). Therefore, it is imperative to implement preventive measures against radiation esophagitis and gastrointestinal adverse effects when patients receive concurrent chemoradiotherapy. Symptoms should be carefully monitored during treatment to avoid long-term complications.

#### 4.2.3 Clinical stage is an influencing factor for symptom clusters

Clinical stage was identified as a significant factor correlated with the dysphagia and energy deficiency SCs by multiple linear regression analysis (Table 3). This finding is consistent with results reported by Zeng et al. (20). TNM stage has been established as an independent prognostic factor for esophageal cancer patients (31, 32). Tumor stages are generally lower and dysphagia and fatigue-pain symptoms are less severe in relation to disease burden in patients with earlier tumor stages, while patients with advanced pathological staging often present with a cachectic appearance. With severe dysphagia from the disease, patients experience substantial weight loss. During treatment, fatigue is a common associated symptom. Consequently, these patients exhibit more severe energy deficiency SC and thus require more supportive care (33). Notably, lesion length has also been identified as an independent prognostic factor associated with survival rates in esophageal cancer (34). This study did not measure lesion length, highlighting the need for clinical nursing staff to comprehensively understand the clinical characteristics of esophageal cancer patients and to develop scientifically systematic symptom cluster prevention programs.

#### 4.2.4 Methodological considerations and integration with dynamic network evidence

In addition to these clinical interpretations, several methodological considerations should be acknowledged. First, the cross-sectional design only reveals statistical covariation among variables and does not permit inferences about temporal order or causality; therefore, all regression results should be interpreted as measures of association strength rather than as predictive or deterministic effects. Second, the low adjusted *R*^2^ values in our models suggest that disease-related factors alone are insufficient to fully account for symptom cluster severity, indicating that future studies should incorporate multidimensional predictors, including biomarkers, psychosocial variables, and dynamic symptom networks, to improve explanatory power.

These limitations also highlight a broader conceptual issue common to most symptom cluster research, including the present study: factor analysis identifies groupings based on single-time-point covariation, but cannot capture how symptoms influence one another over time. A recent longitudinal study by Sun et al. (7) addressed this gap using cross-lagged panel network analysis in esophageal cancer patients undergoing chemotherapy, revealing that pain and dysphagia were the most influential symptoms (highest out-strength centrality) in the early treatment phase, whereas fatigue and nausea became dominant later. Notably, these dynamic findings resonate with our factor-derived clusters—dysphagia and pain co-emerged in our “dysphagia SC,” and fatigue was central to our “energy deficiency SC” alongside sleep disturbance and weight loss. Yet network analysis further demonstrated that these symptoms exerted directional, not merely correlational, effects on others, and that their influence shifted substantially over time—insights that cross-sectional factor analysis cannot provide. Taken together, our results can be viewed as a static structural complement to these dynamic models: factor analysis offers a stable overall grouping of co-occurring symptoms, whereas network analysis adds temporally ordered, probabilistically causal information. Integrating both approaches in future longitudinal designs may therefore yield the most complete picture of symptom dynamics in this population.

## 5 Conclusion

Patients undergoing postoperative chemoradiotherapy for esophageal cancer frequently present with multiple concurrent symptoms that are interrelated and tend to aggregate into distinct clusters. The composition and severity of these clusters are significantly modulated by disease- and treatment-related determinants, such as tumor location, clinical stage, and therapeutic regimen. Clinicians and nursing staff should direct attention to the fundamental disease characteristics that underpin symptom clustering, while placing a strong emphasis on comprehensive symptom appraisal—particularly the interactions among individual symptoms. Strengthening early warning systems and refining the recognition of symptom clusters represent critical priorities. Furthermore, the formulation and implementation of tailored symptom management interventions warrant further investigation in prospective studies to determine their effectiveness in mitigating patient distress, optimizing symptom control, and ultimately enhancing health-related quality of life.

This two-year cross-sectional study was conducted within a single institution; given the cross-sectional design of this study, the causal direction of the aforementioned associations requires validation through future longitudinal or experimental studies. Furthermore, although the sample demonstrates acceptable representativeness, the lack of data from the early treatment phase and the post-treatment follow-up period makes it challenging to comprehensively delineate the longitudinal trajectory and temporal progression of symptom burden throughout the entire treatment course. To address these gaps, future research should adopt a multicenter, large-scale longitudinal study design to assess symptom clusters at different time points and elucidate the dynamic interrelationships among various symptom dimensions over time.

## Data Availability

The original contributions presented in the study are included in the article/Supplementary Material, further inquiries can be directed to the corresponding author/s.
